# Recovery of Diaphragm Function following Mechanical Ventilation in a Rodent Model

**DOI:** 10.1371/journal.pone.0087460

**Published:** 2014-01-27

**Authors:** Christian S. Bruells, Ingmar Bergs, Rolf Rossaint, Jun Du, Christian Bleilevens, Andreas Goetzenich, Joachim Weis, Michael P. Wiggs, Scott K. Powers, Marc Hein

**Affiliations:** 1 Department of Surgical Intensive and Intermediate Care, and Department of Anesthesiology, University hospital of the RWTH Aachen, University of Excellence, Aachen, Germany; 2 Department of cardiothoracic and vascular surgery, University hospital of the RWTH Aachen, University of Excellence, Aachen, Germany; 3 Institute of Neuropathology and JARA – Translational Brain Medicine, University hospital of the RWTH Aachen, University of Excellence, Aachen, Germany; 4 Department of Applied Physiology and Kinesiology, University of Florida, Gainesville, Florida, United States of America; D'or Institute of Research and Education, Brazil

## Abstract

**Background:**

Mechanical ventilation (MV) induces diaphragmatic muscle fiber atrophy and contractile dysfunction (ventilator induced diaphragmatic dysfunction, VIDD). It is unknown how rapidly diaphragm muscle recovers from VIDD once spontaneous breathing is restored. We hypothesized that following extubation, the return to voluntary breathing would restore diaphragm muscle fiber size and contractile function using an established rodent model.

**Methods:**

Following 12 hours of MV, animals were either euthanized or, after full wake up, extubated and returned to voluntary breathing for 12 hours or 24 hours. Acutely euthanized animals served as controls (each n = 8/group). Diaphragmatic contractility, fiber size, protease activation, and biomarkers of oxidative damage in the diaphragm were assessed.

**Results:**

12 hours of MV induced VIDD. Compared to controls diaphragm contractility remained significantly depressed at 12 h after extubation but rebounded at 24 h to near control levels. Diaphragmatic levels of oxidized proteins were significantly elevated after MV (p = 0.002) and normalized at 24 hours after extubation.

**Conclusions:**

These findings indicate that diaphragm recovery from VIDD, as indexed by fiber size and contractile properties, returns to near control levels within 24 hours after returning to spontaneous breathing. Besides the down-regulation of proteolytic pathways and oxidative stress at 24 hours after extubation further repairing mechanisms have to be determined.

## Introduction

Mechanical ventilation (MV) is a common intervention in intensive care units to provide adequate oxygenation of highly sedated patients or with respiratory failure. Although MV is a life-saving intervention, prolonged MV results in diaphragmatic atrophy and contractile dysfunction, which is termed ventilator-induced diaphragmatic dysfunction (VIDD). The mechanism(s) responsible for VIDD continue to be debated but growing evidence reveals that ventilator-induced oxidative stress and protease activation in the diaphragm are major contributors [1]–[3].

As much as 30% of patients treated with prolonged MV experience difficult weaning [4], [5]. Therefore, the failure to wean patients from MV is a significant clinical problem that prolongs time on the ventilator and increases morbidity and mortality [5]. Although the failure to wean may be due to multiple causes as chronic obstructive pulmonary disease (COPD) [6], obesity [7] or cardiac failure [8], VIDD is predicted to be a key contributor [9]. Therefore, it is important to understand how rapidly the diaphragm recovers from VIDD after the return to spontaneous breathing and extubation.

To date, only two studies have examined the impact of reloading the diaphragm (i.e., return to spontaneous breathing) following prolonged MV. Unfortunately, both of these studies were of short duration (i.e., up to 7 hours post MV) and in sedated animals. [10]. The latest investigation primarily focused on the changes in diaphragm contractile function after the first 7 hours of reloading [11]. While the first study did not show a difference to ventilated animals after 2 hours of reloading, the latter reported a nearly complete recovery of contractile function after 7 hours of reloading. Both studies investigated sedated animals breathing via a tracheal tube, which does not mimic the clinical situation of increased workload after extubation. Therefore, it remains unknown how rapidly the diaphragm recovers from VIDD during 12–24 hours following prolonged MV if the diaphragm is fully loaded in awake animals and this forms the rationale for the current experiments.

Thus, using a rat model of prolonged MV, we investigated the time course of diaphragm recovery following 12 or 24 hours of spontaneous breathing. We hypothesized that, following removal from the ventilator and extubation, the return to voluntary breathing would rapidly restore diaphragm muscle fiber size and contractile function. Our results support this hypothesis but indicate that diaphragmatic recovery from VIDD remains incomplete following 12 hours of spontaneous breathing. These findings are important and contribute to our biological understanding of diaphragm recovery from VIDD in the ICU setting.

## Materials and Methods

### Mechanical ventilation protocol

The protocol was approved by the Landesamt für Natur, Umwelt und Verbraucherschutz (Northrhine-Westphalia, Germany (Permit Number: AZ 84-02.04.2011.A277). All surgery was performed under sodium pentobarbital anesthesia, and all efforts were made to minimize suffering.

After induction of anesthesia (Pentobarbital sodium 50 mg/kg body weight intraperitoneally), rats (n = 8 each) were intubated with a 14 G cannula and mechanically ventilated (Physioflex, Evita2, Draeger, Luebeck, Germany). Animals were ventilated using the pressure controlled mode with a positive end-expiratory pressure of 3 cm H_2_O and the lowest peak airway pressure required to maintain normocapnia, breathing frequency was established at 60 breaths/min and inspiratory:expiratory relation of 1∶1. All surgical procedures were performed under sterile conditions, including instruments, catheters and a sterile workplace environment. One specialized tail artery catheter (Rat Femoral Artery Catheter, Braintree Scientific, MA, USA) was placed in the tail artery to record blood pressure and draw blood samples for blood gas analysis. Using a small paramedian incision, the jugular vein was cannulated and used for fluid administration, starting at 1 ml saline per hour, which was changed based on hematocrit values. Blood samples were drawn all three hours to measure arterial gas pressures (paO_2_, paCO_2_) Hemoglobin and hematocrit as well as electrolytes (ABL X, Radiometer, Copenhagen, Denmark). The inspiratory oxygen fraction (FiO_2_) was adapted to maintain pO_2_ between 80 – 120 mmHg. Body temperature was maintained at 37°C using a temperature controlled heating plate. Heart rate was monitored via a lead II electrocardiograph. All vital parameters were recorded using the PowerLab System (AD instruments, Colorado Springs, CO, USA). Anesthesia was maintained using single dose administration of pentobarbital (2 mg i.p., if needed). Anesthetic depth was controlled to suppress breathing efforts by the animal. After completion of 12 h of MV, animals were either euthanized (cardiectomie) (CMV) or extubated.

### Recovery from mechanical ventilation

After completion of 12 h MV time, rats were breathing spontaneously via the tracheal tube. The catheters were removed and the vessels ligated. The tube was removed under suctioning to remove airway mucus after suturing and the airways were intermittently suctioned to remove mucus, if necessary. The animals were placed in a heatable chamber (Vetario S10 Intensive Care Unit, Brinsea, Sandford, UK) and maintained at 37°C until return of full consciousness. After full recovery, the rats were placed back in their cages and taken back to the central animal facility. After 12 or 24 h, anesthesia was reinitiated using Pentobarbital i.p. in the above mentioned dose. At the end of the defined recovery period, animals were intubated under a surgical plane of anesthesia, and put on the ventilator with the above noted settings to prevent hypercapnia and hypoxia before sacrifice; rectal temperature was recorded, the abdomen and chest opened and blood withdrawn via cardiac puncture for laboratory investigations and blood gases. Ventilation time was less than 5 minutes. The diaphragm was removed before cardiectomie.

### Acutely anesthetized controls

Under a surgical plane of anesthesia, animals were intubated and put on the ventilator with the above noted settings to prevent hypercapnia and hypoxia before sacrifice. Ventilation time was shorter than 5 minutes. The diaphragm was removed before cardiectomie.

**Figure 1:**
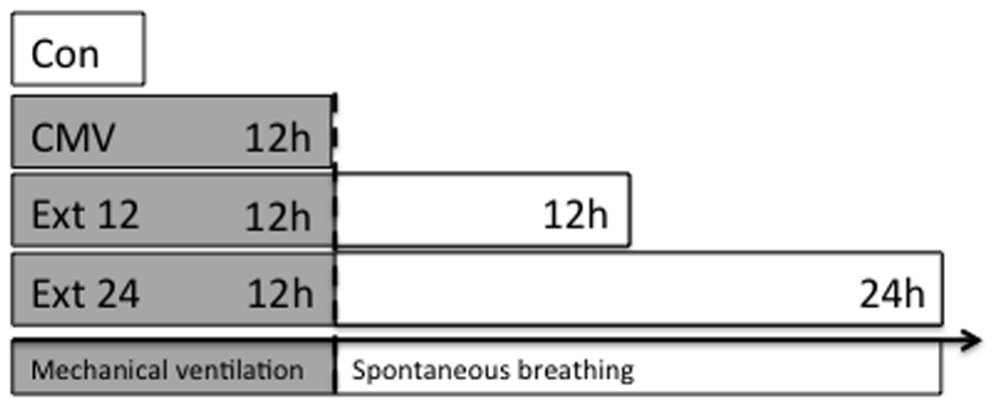
Timeline of the experiments. CMV: Mechanical ventilation for 12 hours, Ext 12: 12 h of spontaneous breathing after 12 hours of MV, Ext 24: 24 h of spontaneous breathing after 12 hours of MV, CON: unventilated controls. The dotted line indicates the end of the mechanical ventilation period.

Please see also FIGURE 1 for a timeline.

### Functional measures

#### Diaphragmatic contractile properties

A muscle strip of around 1.5–2 cm length with tendinous attachments both at the central tendon and rib cage was dissected from the mid-costal region. The strip was suspended vertically using strings with one end connected to an isometric force transducer (FMI Gmbh, Seeheim, Germany) within a jacketed tissue bath filled with Krebs-Henseleit solution at 23°C. After determination of the L0 (optimal muscle length) using single twitches every 30 seconds at maximal stimulator output (Grass S48, Grass technologies, Quincy, MA, USA), force frequency relationships were measured using trains of 300 ms length reaching a force plateau, stimulus delay 0.1 ms and stimulus duration of 2 ms. Contractile force was recorded at 15, 30, 60, 100 and 160 Hz with two minutes rest between contractions[12].

### Histology

#### Fiber cross sectional areas

Sections of diaphragmatic tissue were cut along the fiber orientation and embedded in Tissue Tek (Sakura Finetek Europe, The Netherlands) and frozen in liquid iospentane, which had been precooled in liquid nitrogen at −80°C. Sections were cut at 7 µm using a cryotome, stained for dystrophin protein (rabbit host, #RB-9024- R7, Lab Vision Corporation, Fremont, CA), myosin heavy chain Type I (mouse host, immunoglobulin M (IgM) isotype, A4.840, Developmental Studies Hybridoma Bank, Iowa City, IA, USA), and myosin heavy chain Type IIa (SC-71, mouse host, immunoglobulin G (IgG) Developmental Studies Hybridoma Bank, Iowa City, IA, USA). As secondary antibodies, Rhodamine red, Alexa fluor 350 and Alexa 488 (Invitrogen, Frankfurt, Germany) were used to identify different muscle fiber types. Pictures were made using Fluorescence microscopy (Zeiss Axiovision, Jena, Germany) at 400-fold magnification. Cross sectional area (CSA) was determined using Scion image software counting around 250 fibers per animal (National Institute of Health, Bethesda, MD, USA).

#### Measures of inflammation in diaphragm

Diaphragm strips were stored in 4% buffered formalin directly after dissection. Samples were embedded in paraffin and cut in 7 µm thin slices. After Hematoxilin and Eosin staining, a blinded investigator from the Institute of Neuropathology investigated the slices for invasion of neutrophils and lymphatic cells, using light microscopy in 400 fold magnification. The complete area of the three diaphragm slices was investigated (around 200 fibers per slide).

### Biochemical measures

#### Western blot analysis

We performed Western blots to determine changes in protein levels of AKT, 4-hydroxynonenal and αII-spectrin. A maximum of 30 mg of diaphragm tissue was homogenized on ice in 800 µl lysis buffer containing 150 mM sodium chloride, 1.0% NP-40, 0.1% sodium dodecyl sulfate (SDS), 1% sodium deoxycholate, 50 mM Tris-HCl (pH 7.6; all from Sigma-Aldrich, Germany) and Protease inhibitor cocktail tablets (Roche Diagnostics, Mannheim, Germany), using 2 ml Potter-S homogenization cylinders (Sartorius; Goettingen, Germany). Homogenates were centrifuged through Qiagen-shredder columns (Qiagen, Hilden, Germany) at 2000 rpm for 2 min and the supernatant was used for the determination of protein concentrations, using a DC-Protein Assay Kit (Bio-Rad Laboratories, München, Germany). Samples were boiled for 5 min after addition of Laemmli-buffer (312.5 mM Tris HCl, pH 6.8, 10% SDS, 50% Glycerin, 10% β-Mercaptoethanol, less than 5 mg Bromphenol-blue; all from Sigma-Aldrich). An equal amount of 20 µg of each sample was separated by 10% SDS-Page and transferred onto a PVDF membrane (Bio-Rad Laboratories). After the semi-dry blotting procedure (60 min, 25 V), the membrane was incubated for 1 h at room temperature (RT) in 5% BSA blocking-solution (Albumin fraction V; ROTH, Karlsruhe, Germany), followed by overnight incubation on a shaker at 4°C with specific antibodies against **AKT** (Cell signaling technology, Danvers, MA, USA), **4-hydroxynonenal** (Abcam, Cambridge, UK), and **αII-spectrin** (Santa Cruz Biotechnology, Santa Cruz, CA, USA) and GAPDH as a loading control (Cell signaling technology, Danvers MA, USA). Overnight incubation was followed by repeated washing steps (3×5 min in TBS buffer containing 1% Tween20; Sigma Aldrich) then the membrane was incubated for 1 h at RT on a shaker with horseradish-peroxidase conjugated goat anti-rabbit antibody (#7074; Cell signaling technology). The final reaction was visualized using enhanced chemiluminescence (WEST-ZOL Plus Western blot detection system; iNtRON Biotechnology, Korea) and a detection system (BioDocAnalyze live; biometra, Goettingen, Germany). Images were taken and densitometrically analyzed with the software ImageJ (v1.46 k; National Institute of Health, USA).

### Statistical analysis

Population distribution was assessed with the Shapiro-Wilks test. Comparisons between groups for each dependent variable were made by a one-way analysis of variance (ANOVA) and, for the force frequency measurements using a two-way repeated-measures ANOVA. If the group effect was significant, a Tukey post-hoc test was used for pairwise comparisons between all groups. Data are shown as means ± SD. relationship between variables was assessed with the Pearson correlation coefficient. All statistical tests are two sided, significance was established at p<0.05. (GraphPad Prism 6.0, La Jolla, CA). Power analysis was performed prior to the study assuming an effect size of 1.5 for the reduction of fiber cross sectional area after 12 hours of MV compared to controls with a power of 80%.

## Results

### Systemic and biological response

All animals included in the results survived the experiment with sufficient blood pressure and oxygenation. Four animals did not survive the full duration of MV due to tube blockade and hypoxemia, one animal (EXT24) was excluded for unnatural behavior and increased body temperature and was replaced. One animal in the CMV group died at the end of the experiment and was not replaced: data of CMV group pertains to 7 animals. Four animals suffered from respiratory failure after extubation due to insufficient mucus clearance and had to be euthanized and were replaced. The weight before the experiment did not differ between the animals. Further parameters are displayed in tables 1 and 2.

**Table 1:** Arterial blood gases, blood pressure, Temperature of the animals investigated during the experiment.

	CON	CMV 6 h	EXT 12 6 h	EXT 24 6 h
PaO_2_ (mmHg)	122±9.7	106±13^+^	136±24	127±24
PaCO_2_ (mmHG)	26±10	40±9	40±12	35±12
SpO_2_ (%)	95±2	95±2	96±2	96±1
Mean BP (mmHg)	n.a.	128±29	137±14	134±10
Core Temp (°C)	36.6±0.4	36.5±0.3	36.9±0.3	36.4±0.9
Body weight (g)	298±12.2			
pH	7.53±0.13	7.35±*0.12	7.42±0.07	7.43±0.1

CMV 12: Mechanical ventilation for 12 hours, Ext 12: 12 hours of spontaneous breathing after 12 hours of MV, Ext 24: 24 hours of spontaneous breathing after 12 hours of MV, CON: unventilated controls. n.a. not applicable. BP: blood pressure. Body weight determined before sacrifice. + p<0.05 CVM vs Ext 12. * p<0.05 Con vs CMV. Data are mean ± SD.

**Table 2:** Arterial blood gases, blood pressure and temperature of the animals investigated at the end of the experiment.

	CON	CMV 12 h	EXT12 12 h	EXT 24 24 h
PaO_2_ (mmHg)	122±9.7	126±31	116±37	102±30
PaCO_2_ (mmHG)	26±10	33±8	21±6^+^	22±5
SpO_2_ (%)	95±2	97±1	96±2	96±2
Mean BP (mmHg)	n.a.	125±32	n.a.	n.a.
Core Temp (°C)	36.6±0.4	36.5±0.4	36.5±0.4	36.2±0.5
Body weight (g)	298±12.2	272.8±28.3	301.9±19.4	289±14.39
pH	7.53±0.13	7.50±0.1	7.6±0.13	7.55±0.1

CMV 12: Mechanical ventilation for 12 hours, Ext 12: 12 hours of spontaneous breathing after 12 hours of MV, Ext 24: 24 hours of spontaneous breathing after 12 hours of MV, CON: unventilated controls. n.a. not applicable. BP: blood pressure. Body weight determined before sacrifice. + indicates p<0.05 CMV vs Ext 12. Data are mean ± SD.

### Diaphragm contractile dysfunction and atrophy

**Figure 2:**
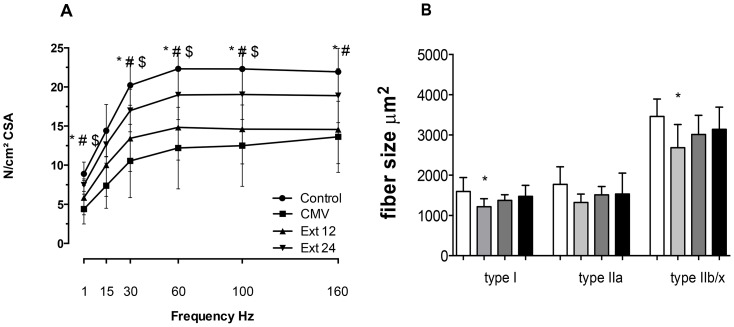
Comparison of diaphragm contractile force (A) and diaphragm fiber size (B) across the experimental groups. A) Force-frequency curves for diaphragm B) Fiber cross-sectional area analysis (CSA) of diaphragm sections stained for dystrophin, myosin heavy chain (MHC) I and MHC type IIa proteins. Key: CMV: Mechanical ventilation for 12 hours (n = 7), light grey bar; Ext 12: 12 h of spontaneous breathing after 12 hours of MV (n = 8), dark grey bar; Ext 24: 24 h of spontaneous breathing after 12 hours of MV (n = 8), black bar; CON: unventilated controls (n = 8), white bar. Values are mean ± SD. * significant CMV vs control p<0.05; # significant Ext 12 vs control p<0.05, $ significant CMV vs Ext 24.

Twelve hours of MV resulted in a significant decrease in diaphragm contractile function. Diaphragm contractile function remained significantly reduced 12 hours after extubation but improved to levels not different from control at 24 hours of spontaneous breathing, (see FIGURE 2, A). Furthermore, compared to control, 12 hours of MV resulted in a significant decrease in diaphragm fiber size in type I and IIb fibers. Following extubation and the return to spontaneous breathing, diaphragm fiber size recovered progressively and did not differ from control or CMV animals following 12 and 24 hours of recovery (FIGURE 2, B).

### Inflammatory response

Histological examination of 7 µm sections (3 different sections per animal) of diaphragm muscle revealed no detectable invasion of neutrophils or lymphatic cells around diaphragm muscle fibers in any experimental group. We interpret this observation as evidence that 12 hours of MV in our model of healthy rodents and subsequent recovery from MV is not associated with a cellular inflammatory reaction.

### Biomarkers of anabolic signaling, oxidative stress, and protease activity

**Figure 3:**
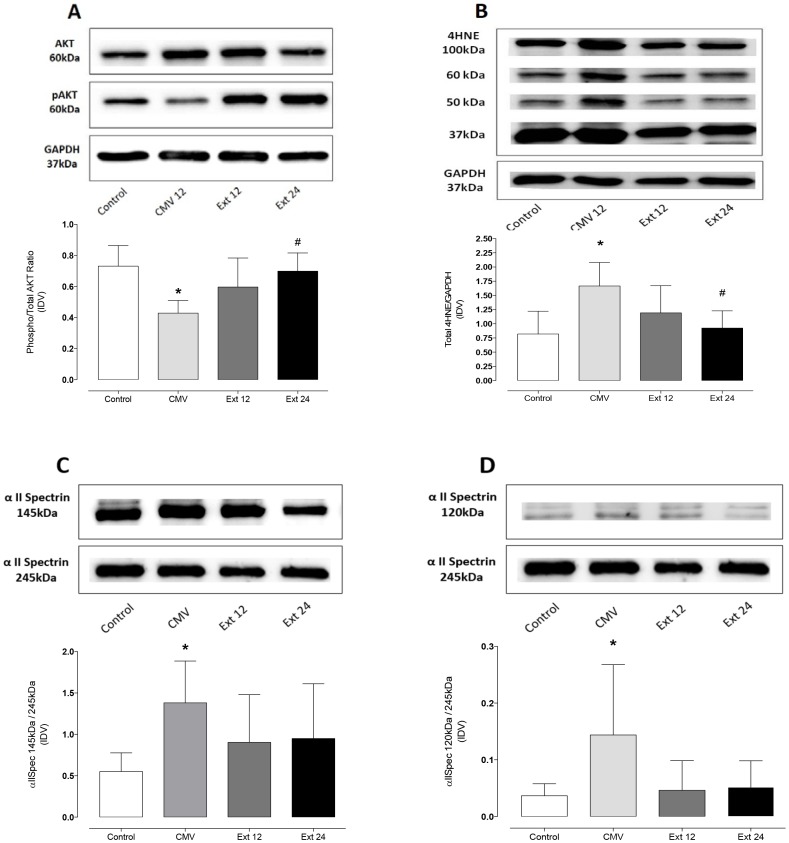
Western blot results of biomarkers of protein synthesis, proteolysis and oxidative stress in the diaphragm. A) pAKT/AKT ratio was analyzed as quotient of pAKT versus AKT expression. Representative blots for both AKT and pAKT are displayed above; Con n = 8, CMV n = 6, Ext 12 n = 8, Ext24 n = 8 B) The levels of 4-hydroxynonenal (4- *HNE*) were analyzed as an indicator of lipid peroxidation via Western blotting. A representative blot for 4-HNE protein conjugates is shown above the graph (Con n = 8, CMV n = 7, Ext 12 n = 8, Ext24 n = 8). C/D) Calpain (C) (Con n = 6, CMV n = 7, Ext 12 n = 7, Ext24 n = 8) and caspase-3 (D) (Con n = 7, CMV n = 7, Ext 12 n = 7, Ext24 n = 8) activations were determined via Western blotting as a ratio for the spectrin breakdown product (120 and 145 kDA band) compared to the 245 kDa band. Representative Western blots are shown above the graphs. CMV 12: Mechanical ventilation for 12 hours, Ext 12: 12 hours of spontaneous breathing after 12 hours of MV, Ext 24: 24 hours of spontaneous breathing after 12 hours of MV, CON: unventilated controls. Values are arbitrary units ± SD. * significant versus Control, # significant versus CMV; IDV integrated density value.

We measured diaphragmatic levels of the ratio of Phospho-AKT/AKT as an index of anabolic signaling. Diaphragm levels of the ratio of Phospho-AKT/AKT were significantly reduced after 12 hours of CMV (p<0.002). Following extubation, diaphragm Phospho-AKT/AKT levels were restored to control levels at 24 hours of recovery (p = 0.006) (FIGURE 3A). Absolute levels of AKT were not changed significantly.

As a biomarker of diaphragm oxidative damage, we determined diaphragm levels of 4-HNE-conjugated proteins via western blotting. Compared to controls, diaphragmatic levels of 4-HNE were significantly increased (p<0.002) after CMV but returned to baseline at 24 hours during recovery (p = 0.007)(FIGURE 3B).

Because both calpain and caspase-3 have been shown to play a major role in VIDD, we measured diaphragmatic levels of specific degradation products of αII-spectrin for both proteases calpain (145 kDa) and caspase 3 (120 kDa) as ratio to the uncleaved product (245 kDa) as biomarkers of protease activity. Twelve hours of CMV resulted in significant increases in diaphragmatic levels of the specific breakdown product of calpain (145 kDa, p = 0.04) and caspase 3 (120 kDa, p = 0.04) indicating increased activity of these key proteases in the diaphragm during CMV. Following extubation, ratio of diaphragmatic levels of αII-spectrin cleavage products for calpain (145 kDA) and caspase 3 (120 kDa) did not differ significantly from control levels or CMV (FIGURES 3C and 3D).

### Correlation between contractile force and the other measures

To further determine whether contractile force was linked to the level of oxidized proteins or to some markers of atrophy pathways, correlations between diaphragm function and these different measures were investigated. These data showed that the levels of oxidized proteins were significantly and negatively correlated with the diaphragm force at all stimulation frequencies while the type IIx/b fiber size was positively correlated with the diaphragm force at all frequencies as was the ratio of pAKT/AKT at 30 Hz (see table 3).

**Table 3:** Pearson correlations (R) between diaphragm specific contractile force and further parameters influenced during mechanical ventilation.

Dependent measure		15 Hz	30 Hz	60 Hz	100 Hz	160 Hz
4-HNE	Pearson Correlation	**R = −.665**	**R = −.621**	**R = −.608**	**R = −.594**	**R = −.524**
	p-value	**0.001**	**0.001**	**0.001**	**0.001**	**0.004**
pAkt/Akt	Pearson Correlation	**R = **.326	R = .381^*^	**R = **.365	**R = **.353	**R = **.336
	p-value	0.091	**0.045**	0.056	0.065	0.081
Type IIb fiber CSA	Pearson Correlation	R = .447^*^	R = .423^*^	R = .425^*^	R = .433^*^	R = .456^*^
	p-value	**0.015**	**0.022**	**0.022**	**0.019**	**0.013**

Pearson correlations (R) between diaphragm specific contractile force production (all stimulation frequencies) and 4-HNE concentration, pAkt/Akt ratio, and the cross sectional area of diaphragm type IIb fibers. 4HNE: 4-Hydroxy-non-enal, AKT = Proteinkinase B, CSA: Cross-sectional area.

## Discussion

### Overview over principle findings

These experiments provide new and important information regarding the time course and biological response of the diaphragm recovery from VIDD. Our results support the prediction that diaphragmatic recovery from VIDD is relatively rapid as biomarkers of oxidative stress, anabolic signaling, and protease activity in the diaphragm returned to control levels during 12 hours of spontaneous breathing following MV. Nonetheless, diaphragmatic recovery, as indexed by force production, remains incomplete during the first 12 hours following MV while complete recovery regarding diaphragm force and other markers was observed after 24 hours of spontaneous breathing. A brief discussion of these results follows.

### Diaphragmatic recovery from VIDD

These are the first experiments to investigate the time course of diaphragmatic recovery during the first 24 hours following prolonged MV using a model of extubation and fully awake animals. Our results reveal that the MV-induced atrophy in type I, IIa and type IIx/b fibers was gradually corrected during the 24 hour recovery period returning to control levels at 12 hours following extubation. In contrast, diaphragmatic specific force production remained depressed below controls at 12 hours during the recovery period.

Our finding that diaphragmatic contractile properties remain depressed for 12 hours following reloading differs from the work of Thomas et al. [11] who observed complete recovery of diaphragm contractile properties at 4–7 hours of recovery following 24 hours of MV. The explanation for these divergent findings is unclear. Importantly, our model combines effects of re-activation of the diaphragm, which ameliorates the activation proteolytic cascade and, in the same time leads to an uncontrolled loading of the diaphragm. Overloading of the diaphragm leads to injury itself [13] and may be existent in our model. Regardless, it seems likely that the mechanism(s) responsible for MV-induced reduction in diaphragmatic specific force production is likely due to several changes in diaphragm muscle fibers including oxidative damage to myofibrillar proteins [14] and proteolytic cleavage of sarcomeric structural proteins resulting in a reduced ability for the sarcomere to generate force [1], [15]. In this regard, our data reveal that 24 hours of recovery from MV result in the disappearance of 4-HNE-conjugated proteins (biomarker of oxidative damage) in the diaphragm and close correlation between the levels of 4-HNE conjugated proteins and diaphragmatic force in all frequencies. With this reduction in oxidized proteins, we do observe a significant increase in force production after 24 hours of recovery compared to CMV. Importantly, Thomas and colleagues could not detect a decrease in oxidative damage after 4–7 hours of spontaneous breathing although recovery of function occurs [11]. Both studies suggest that oxidative modification of diaphragm proteins is a major, but not solely the explanation for the contractile deficit after MV.

### Inflammatory reaction to mechanical ventilation and reloading

Our results do not reveal increases in cellular inflammatory reaction (i.e. macrophage or neutrophil or lymphatic invasion) around diaphragm fibers at any time point during recovery, which is consistent with the findings after 24 hours of MV [10]. Interestingly, 2 hours of reloading led to an infiltration of neutrophils [10], which might have disappeared after 12 hours of reloading. Thomas and colleagues revealed an increase of neutrophils after 4–7 hours after reloading, but no increase in macrophage invasion. In contrary, resistive breathing leads to invasion of inflammatory cells with a peak after 3 days after intermediate resistive loading [16]. We are aware that in our study two processes may occur, i.e. a decrease in inflammation due to the return of spontaneous breathing vs. an increase of diaphragmatic load that may induce inflammation, too. It is possible that the time span chosen in our experiment (i.e. 12 hours and 24 hours of reloading) may miss an inflammatory response of both processes.

### Changes in anabolic signaling and proteolysis in the diaphragm during recovery from VIDD

We measured diaphragmatic levels of active AKT as a biomarker of anabolic signaling. Indeed, active (phosphorylated) AKT plays a key role in promoting protein synthesis in skeletal muscle by regulation of mammalian Target of Rapamycin (mTOR) signaling and protein translation [17], [18]. Moreover, active AKT depresses muscle proteolysis by preventing FOXO3a-induced expression of key ubiquitin-proteasome proteins and required autophagy proteins [3]. It follows that decreases in the pAKT/AKT ratio promotes proteolysis via FOXO3a signaling during muscle disuse [17]. Our results reveal that 12 hours of prolonged MV results in decreased active AKT in the diaphragm, which is linked to a decrease in diaphragm protein synthesis and accelerated proteolysis. This ventilator-induced decrease in active AKT rebounded during recovery and reached control levels by 24 hours following MV.

Identical to previous reports, our results indicate that 12 hours of MV activates calpain and caspase-3 in the diaphragm [1], [19], [20]. This is significant because this proteases play a required role in the development of VIDD and both are closely linked to each others' action [21]. Consistent with our observation of diaphragmatic recovery during reloading, the degradation products of both proteases decreased during recovery and did not differ from control levels following 12/24 hours of spontaneous breathing. Thomas and colleagues could not find a difference between ventilated animals and reloaded animals after breathing in sedated state for 4–7 hours, which might be due to reduced reloading of the diaphragm [11]. However, the decrease of proteolytic action as shown in our study may allow the re-establishment of cellular homeostasis of protein synthesis and breakdown.

### Clinical implications and experimental limitations

We selected the rat as the animal model in the current study because of the anatomical and biochemical similarities between the rat and human diaphragm [22], [23]. Importantly, a recent review concludes that the structural and biochemical changes that occur in the rat diaphragm during prolonged MV are indistinguishable to the changes that occur in humans diaphragm during prolonged MV [9]. Hence, it is feasible that our results using this animal model of MV could be predictive of the time course required for recovery of the human diaphragm from VIDD.

Nonetheless, it is important to note that our experiments were performed in lean and healthy animals. Therefore, it is unclear if our results accurately predict the time course of diaphragm recovery in human patients with co-morbidities such as obesity, diabetes, heart failure, sepsis, or other disorders. Moreover, ICU patients may display diaphragmatic weakness before MV is initiated [24]–[26], where VIDD serves as ‘second hit’ to an already impaired diaphragm. Recovery time of these patients might be slower compared to our data from healthy animals. Further, because our experimental animals were young adults, our results cannot predict the time course of diaphragm recovery from MV in senescent animals. Indeed, age is likely an important factor that determines the rate of diaphragm recovery since previous reports show that limb muscle in aged animals recovers from injury or disuse atrophy at a slower rate than muscles from young adult animals [27]. Additional studies are required to determine the impact of disease and/or age on diaphragm recovery from MV.

Finally, our diaphragm recovery study was performed during the first 24 hours following 12 hours of continuous MV. While 12 hours of MV results in a significant level of VIDD, previous studies reveal that longer periods of MV result in even greater levels of diaphragmatic atrophy and contractile dysfunction [28]. Therefore, the current results may not reflect the time course of diaphragm recovery following long durations (e.g. days to weeks) of MV. How rapidly the diaphragm recovers from extremely long periods of MV is an important issue that warrants further research. At the completion of the experiment, our 12 animals had a significantly lower PaCO_2_ compared to CMV animals. Nonetheless, these levels of PaCO_2_ do not depress diaphragmatic contractility [29].

## Conclusions

These are the first experiments to investigate diaphragmatic recovery during the first 24 hours following MV. Our results reveal that the diaphragm recovers rapidly during the first 24 hours following MV. Nonetheless, diaphragm force remains depressed at 12 hours after the termination of MV but recovered progressively after 24 hours of spontaneous breathing.

This failure of diaphragm function to completely recover from MV within the first 12 hours of the return to spontaneous breathing may explain, at least in part, the difficulty to prevent extubation failure in some patients after MV. Indeed, it is predicted that the failure to recover from VIDD could contribute to difficult weaning particularly in patients with an increased work of breathing (e.g., COPD patients or obese patients).

Finally, future studies should investigate longer durations of recovery using a combination of weaning strategies in an effort to an optimal strategy to promote diaphragm recovery from VIDD.
